# Association of pulsatile stress in childhood with subclinical renal damage in adults: A 30‐year prospective cohort study

**DOI:** 10.1111/jch.14360

**Published:** 2021-09-08

**Authors:** Yueyuan Liao, Chao Chu, Yang Wang, Wenling Zheng, Qiong Ma, Jiawen Hu, Yu Yan, Jun Yang, Ruihai Yang, Keke Wang, Yue Yuan, Chen Chen, Yue Sun, Yuliang Wu, Jianjun Mu

**Affiliations:** ^1^ Department of Cardiovascular Medicine First Affiliated Hospital of Xi'an Jiaotong University Xi'an China; ^2^ Key Laboratory of Molecular Cardiology of Shaanxi Province Xi'an China; ^3^ Institute of Cardiovascular Sciences Hanzhong People's Hospital Hanzhong China; ^4^ Department of Cardiovascular Medicine Jiangsu Province Hospital Nanjing China

**Keywords:** adulthood, childhood, cohort study, pulsatile stress, subclinical renal damage

## Abstract

The pulsatile stress in the microcirculation may contribute to development or progression of chronic kidney disease. However, there is no prospective data confirming whether pulsatile stress in early life affect renal function in middle age. The authors performed a longitudinal analysis of 1738 participants aged 6–15 years at baseline, an ongoing Adolescent Prospective Cohort with a follow‐up of 30 years. The authors evaluated the association between pulsatile stress in childhood and adult subclinical renal damage (SRD), adjusting for related covariates. Pulsatile stress was calculated as resting heart rate × pulse pressure. Renal function was assessed with estimated glomerular filtration rate (eGFR) and urine albumin‐to‐creatinine ratio (uACR). The results showed that pulsatile stress in childhood was associated with adult SRD (Relative Risk, 1.43; *p *= .032), and the predictive value of combined pulse pressure and heart rate for SRD was higher than either of them alone. The high pulsatile stress in childhood increased the risk of adult SRD in males (RR, 1.92; *p *= .003), but this association was not found in females (RR, 0.91; *p *= .729). Further, the participants were categorized into four groups on the basis of pulsatile stress status in childhood and adulthood. Male patients with high pulsatile stress during childhood but normal pulsatile stress as adults still had an increased risk of SRD (RR, 2.04; 95% CI, 1.18–3.54), while female patients did not (RR, 0.96; 95% CI, 0.46–1.99). The study demonstrated that high pulsatile stress in childhood significantly increased the risk of adult SRD, especially in males. Adequate control of pulse pressure and heart rate from childhood, in the long‐term, is very important for preventing kidney damage.

## 1 INTRODUCTION

The chronic kidney disease (CKD) has become an important public health problem in the world.^1^ It has been reported that the prevalence of adult CKD was 10.8% in China and 13.0% in the United States.^2^, ^3^ Hemodynamic studies have shown that if the resistance of the glomerular efferent arterioles is higher than that of the afferent arterioles, the glomerular capillaries are in a high pressure pulsatility. The high pulsatile stress (PS) can distort the material of the glomerular arterial wall,^4^ affect the normal structure and function of renal microvascular, and lead to the occurrence and progression of CKD.^5^, ^6^

Many previous studies have shown that pulsatile stress, may lead to strained elastic fibers, followed by smooth muscle cell hypertrophy, proliferation, and, eventually, arterial stiffness.^7^, ^8^, ^9^ And the change of pulsatile stress was a good predictor of arterial stiffness over time.^10^ Thomas and coworkers investigated 125 513 men and 96 301 women aged 16–95 years and observed that a combined elevation of pulse pressure (PP) and heart rate (HR) increased the risk of cardiovascular mortality in men.^10^ In addition, the Strong Heart Study also reported that pulsatile stress was very important in causing atherosclerosis and vascular hypertrophy.^11^

Direct effects of the pulsatile stress on heart, arterial stiffness, and ultimately cardiovascular survival have been documented whereas little data exist relating to renal damage, especially in adolescents. Therefore, the aim of this study was to investigate the association between pulsatile stress in childhood and adult subclinical renal damage in an ongoing adolescent prospective cohort.

## 2 MATERIALS AND METHODS

### 2.1 Study participants

This study is based on the Hanzhong Adolescent Hypertension Cohort, an ongoing prospective cohort study. The study began in 1987, when 4623 schoolchildren aged 6–15 years who had no chronic diseases in their medical history and who could communicate normally in Mandarin were recruited into the cohort from 26 rural sites of three towns (Qili, Laojun, and Shayan) in Hanzhong, Shaanxi, China. Later, information was collected in 1989, 1992, 1995, 2005, and 2013. The most recent follow‐up of our cohort was in 2017, with a maximum follow‐up of 30 years and a follow‐up rate of 60.1% (*n* = 2780). The reasons for loss of follow‐up mainly included death, mental illness, military service, and migration. The detailed study design and procedures have been published previously.^12^, ^13^, ^14^ Individuals who were pregnant or lactating women, had no blood samples and/or missing measurements and were unable to provide informed consent at follow‐up were excluded, and finally, 1738 individuals were included in the analysis. The participant selection process is described in Figure S1.

This study followed the principles of the Helsinki Declaration. This study was approved by the Academic Committee of the First Affiliated Hospital of Xi'an Jiaotong University (XJTU1AF2015LSL‐047) and was clinically registered (NCT02734472). All participants in this study signed informed consent forms at baseline and during follow‐up. For participants younger than 18 years of age at baseline, informed consent from a parent/guardian was obtained.

### 2.2 Data collection

Personal basic information, family medical history, smoking status, and alcohol consumption history were collected using a unified questionnaire by trained staff. Height, body weight, hip and waist circumferences, and bust size were measured with the participants in underwear and without shoes using appropriate instruments and a uniform standard. Two measurements of these indicators were performed, and the mean values were used for analysis. Body mass index (BMI) was calculated as kilograms per square meter (kg/m^2^).

Seated blood pressure (BP) was measured in a quiet environment by trained and certified staff according to the procedures recommended by the WHO. A Hawksley random zero sphygmomanometer was used for the first six visits, and an Omron M6 (Omron, Kyoto, Japan) device was used in 2017 for BP measurements. Participants were required to avoid coffee/tea, alcohol, cigarette smoking, and strenuous exercise for at least 30 min before the BP measurement. An appropriate cuff size was used. BP was measured three times, with an interval of 2 min between each measurement, and the BP levels were defined as the mean values of the three BP measurements. Pulse measurements in 1987 were performed manually by trained professional medical staff. After these pediatric patients had rested for 5 min in a quiet environment, their pulse times was measured manually by our staff during 30 s. The value is then multiplied by two to obtain an individual's heart rate. At follow‐up, the individuals’ heart rate was obtained simultaneously by Omron sphygmomanometer. Pulse pressure was calculated as systolic BP minus diastolic BP. Pulsatile stress was calculated as resting heart rate × pulse pressure. We determined the pulsatile stress quartiles according to sex‐specific and age‐specific population, and considered the 4th quartile as high pulsatile stress.

### 2.3 Biochemical parameter measurements

Fasting venous blood samples were obtained by experienced nurses in the morning after the participants had fasted for 8–10 h. A Hitachi 7060 automatic biochemical analyzer was used to detect the serum biochemical parameters, including fasting glucose, serum creatinine, uric acid (UA), total cholesterol (TC), triglycerides (TGs), LDL cholesterol (LDL‐C), and HDL cholesterol (HDL‐C). Urinary UA, creatinine, and albumin levels were evaluated with an automatic biochemical analyzer. Details about have been described previously.^15^, ^16^

### 2.4 Assessment of renal function

Renal function was assessed with estimated glomerular filtration rate (eGFR) and urinary albumin creatinine ratio (uACR).^1^ eGFR was calculated using the formula as follows: eGFR = 175×serum creatinine^−1.234^ ×age^−0.179^ (×0.79 for girls/women), where serum creatinine concentration is in milligrams per deciliter and age is in years.^2^ ^17^ uACR was calculated as urine albumin in milligrams divided by the urine creatinine in millimole (milligrams per millimole). Presence of SRD was defined as eGFR between 30 and 60 ml/min per 1.73 m^2^ ^18^ or elevated uACR of at least 2.5 mg/mmol in men and 3.5 mg/mmol in women^19^ as previously described.

### 2.5 Statistical analyses

Continuous variables were shown as mean ± SDs if normally distributed or median (quartile 1, quartile 3) if non‐normally distributed. Categorical variables were expressed as numbers and percentages of patients. Statistical analysis was performed by t test when normally distributed; otherwise, the Mann–Whitney U test was used. Differences between groups of categorical variables were compared with chi‐square tests. Correlation analysis was determined with the Pearson correlation coefficient or the Spearman correlation coefficient when appropriate. Logistic regression analysis was used to determine the association between pulsatile stress in childhood and SRD in adults. Strengths of associations were determined by estimating the relative risks (RR) and their 95% confidence intervals (95%CI). Furthermore, we conducted a sensitivity analysis by excluding individuals who had diabetes or received antihypertensives therapy (*n* = 82) to eliminate the influence of these factors on the results. All statistical analyses were performed using SPSS 25.0 (SPSS, Inc., Chicago, Illinois, USA). Statistical significance was set as a two‐tailed *p* value of less than .05.

## 3 RESULTS

### 3.1 Participants’ clinical characteristics

**FIGURE 1:**
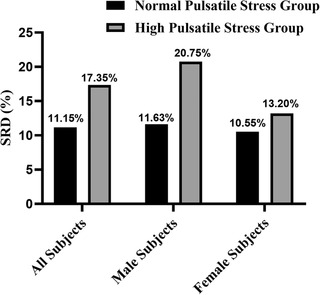
Prevalence of adult subclinical renal damage in normal pulsatile stress group and high pulsatile stress group, overall and by sex group. SRD, subclinical renal damage

The participants’ clinical characteristics in childhood and adulthood by sex are summarized in Table 1. In childhood, female patients had higher body mass index (BMI), busts, systolic BP, diastolic BP, and heart rate than male patients. The pulsatile stress was 2936.0 (2470.9, 3469.2) in all patients, 2933.6 (2448.0, 3432.0) in male patients and 2941.2 (2520.0, 3520.0). The Pulsatile stress quartiles of study participants in childhood according to the age categories are shown in Table S1. In adulthood, male patients had higher height, weight, BMI, waist, hips, systolic BP, diastolic BP, pulse pressure, triglycerides, total cholesterol (TC), low‐density lipoprotein cholesterol (LDL‐C), fasting glucose, serum uric acid, serum creatinine, urine albumin and urine uric acid than female patients, and females had higher heart rate, high‐density lipoprotein cholesterol (HDL‐C), eGFR and uACR than male patients. Males were more likely to smoke and drink than females. The pulsatile stress was 3268.0 (2859.7, 3797.2) in all patients, 3252.7 (2852.0, 3792.0) in male patients and 3290.0 (2871.3, 3812.7). The prevalence of adult SRD in normal pulsatile stress (NPS) group and high pulsatile stress (HPS) group are shown in Figure 1. The prevalence of adult SRD in NPS group and HPS group were 11.15% and 17.35% in all patients, 11.63% and 20.75% in male patients, 10.55% and 13.20% in female patients.

**TABLE 1:** Demographic and clinical characteristics of the study participants

Variable	All patients	Male patients	Female patients	*p* value
No. of patients	1738	963 (55.4%)	775 (44.6%)	
Childhood				
Age (years)	12 (9, 14)	12 (9, 14)	12 (9, 14)	.256
Height (cm)	136.8 (124.0, 148.6)	135.5 (123.5, 148.0)	138.4 (124.2, 149.2)	.088
Weight (kg)	29.5 (23.0, 38.9)	28.8 (23.0, 37.5)	30.9 (23.0, 40.0)	.028
BMI (kg/m^2^)	15.9 (14.8, 17.7)	15.8 (14.8, 17.2)	16.1 (14.7, 18.0)	.030
BMI z‐score	−0.78 (−1.33, −0.25)	−0.82 (−1.42, −0.28)	−0.74 (−1.27, −0.21)	.01
Busts (cm)	61.5 (57.0, 68.0)	61.1 (56.0, 67.9)	61.5 (58.0, 69.0)	.001
Systolic BP (mm Hg)	102.6 (96.0, 110.0)	101.3 (95.3, 110.0)	103.3 (97.0, 110.0)	.017
Diastolic BP (mm Hg)	64.0 (59.3, 70.7)	63.3 (59.0, 70.7)	64.7 (60.0, 70.7)	.022
Pulse Pressure (mm Hg)	38.0 (32.0, 43.3)	37.3 (32.0, 43.4)	38.0 (32.0, 43.3)	.598
Pulsatile stress (aU)	2936.0 (2470.9, 3469.2)	2933.6 (2448.0, 3432.0)	2941.2 (2520.0, 3520.0)	.071
HR (bpm)	78.0 (72.0, 84.0)	78.0 (72.0, 84.0)	80.0 (72.0, 84.0)	.002
Adulthood				
Age (years)	42 (39, 44)	42 (39, 44)	42 (39, 44)	.256
Height (cm)	163.2 (156.8, 168.5)	167.6 (164.0, 171.5)	156.8 ± 5.6	<.001
Weight (kg)	63.3 (55.8, 71.4)	69.0 ± 9.9	56.9 (52.4, 62.9)	<.001
BMI (kg/m^2^)	23.9 (21.9, 26.1)	24.5 ± 3.1	23.2 (21.5, 25.2)	<.001
Waist (cm)	84.5 (78.0, 91.5)	87.7 ± 9.1	80.7 (75.5, 86.7)	<.001
Hips (cm)	92.3 (89.0, 95.7)	92.9 ± 5.4	91.5 (88.4, 95.2)	<.001
Systolic BP (mm Hg)	121.3 (112.3, 131.0)	124.7 (116.3, 133.3)	116.7 (108.0, 126.7)	<.001
Diastolic BP (mm Hg)	76.0 (69.0, 84.0)	78.7 (72.3, 86.3)	72.7 (65.7, 79.7)	<.001
Pulse Pressure (mm Hg)	44.7 (40.3, 50.6)	45.3 (41.0, 51.0)	44.0 (39.3, 50.0)	.002
Pulsatile stress (aU)	3268.0 (2859.7, 3797.2)	3252.7 (2852.0, 3792.0)	3290.0 (2871.3, 3812.7)	.191
HR (bpm)	73.0 (67.0, 80.0)	72.0 (65.0, 78.0)	75.0 (68.0, 82.0)	<.001
Smoking (%)	755 (43.4%)	731 (75.9%)	24 (3.1%)	<.001
Drinking (%)	508 (29.2%)	463 (48.1%)	45 (5.8%)	<.001
FH.hypertension (%)	908 (52.2%)	487 (50.6%)	421 (54.3%)	.120
FH.diabetes (%)	278 (16.0%)	142 (14.7%)	136 (17.5%)	.113
Fasting glucose (mmol/l)	4.57 (4.28, 4.91)	4.59 (4.29, 4.93)	4.55 (4.26, 4.87)	.049
TC (mmol/l)	4.49 (4.03, 4.99)	4.53 (4.04, 5.06)	4.46 (4.0, 4.91)	.009
HDL‐C (mmol/l)	1.14 (0.99, 1.33)	1.07 (0.94, 1.22)	1.24 (1.08, 1.43)	<.001
LDL‐C (mmol/l)	2.50 (2.12, 2.89)	2.56 (2.18, 2.99)	2.42 (2.05, 2.78)	<.001
Triglycerides (mmol/l)	1.35 (0.96, 1.94)	1.52 (1.09, 2.17)	1.16 (0.85, 1.63)	<.001
Serum uric acid (μmol/L)	278.8 (225.3, 335.1)	320.1 (280.7, 369.3)	227.0 (196.4, 266.6)	<.001
Urine uric acid (μmol/L)	1309.5 (936.0, 2015.5)	1357.0 (986.0, 2158.5)	1264.0 (872.0, 1824.0)	<.001
Serum creatinine (μmol/L)	75.6 (66.4, 86.0)	83.4 (75.1, 91.1)	67.3 (60.5, 73.5)	<.001
Urine albumin (mg/L)	7.65 (4.10, 13.90)	8.5 (4.8, 14.3)	6.4 (3.3, 13.1)	<.001
uACR (mg/mmol)	0.97 (0.63, 1.73)	0.88 (0.60, 1.57)	1.11 (0.69, 1.98)	<.001
eGFR (ml/min per 1.73 m^2^)	97.66 (87.21, 111.25)	96.1 (85.9, 109.7)	99.7 (89.2, 113.5)	<.001

Continuous variables were shown as mean ± SDs if normally distributed or median (quartile 1, quartile 3) if non‐normally distributed. Categorical variables were expressed as numbers and percentages of patients. Statistical analysis was performed by *t* test when normally distributed; otherwise, the Mann–Whitney *U* test was used. Differences between groups of categorical variables were compared with chi‐square tests.

*Abbreviations*: BMI, body mass index; BP, blood pressure; eGFR, estimated glomerular filtration rate; FH.hypertension, family history of hypertension; FH.diabetes, family history of diabetes; HR, heart rate; HDL‐C, high‐density lipoprotein cholesterol; LDL‐C, low‐density lipoprotein cholesterol; TC, total cholesterol; uACR, urinary albumin‐to‐creatinine ratio.

### 3.2 Association between pulsatile stress in childhood and adult SRD

**FIGURE 2:**
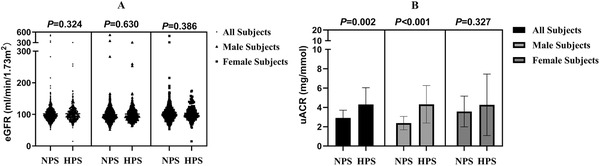
The levels and differences of the eGFR (A) and uACR (B) in normal pulsatile stress group and high pulsatile group, overall and by sex group. eGFR, estimated glomerular filtration rate; uACR, urinary albumin‐to‐creatinine ratio; NPS, normal pulsatile stress group; HPS, high pulsatile stress group

The differences of eGFR and uACR between NPS group and HPS group are shown in Figure 2. There were no differences in eGFR values between NPS group and HPS group, regardless of sex (all *p *> .05). The HPS group had higher uACR values than NPS group (*p *= .002). In male patients, HPS group had higher uACR values than NPS group (*p *< .001), but there was no difference between the two groups in female patients (*p *= .327).

**FIGURE 3:**
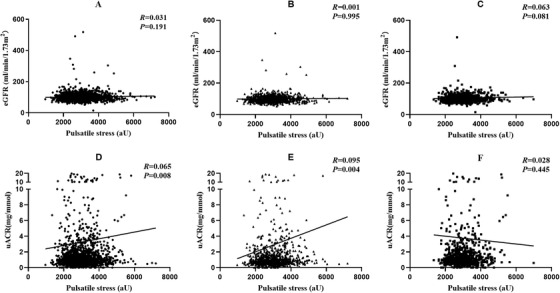
Relationship between pulsatile stress and eGFR and uACR by correlation analysis. Relationship between pulsatile stress and eGFR in overall patients (A), male patients (B) and female patients (C). Relationship between pulsatile stress and uACR in overall patients (D), male patients (E) and female patients (F). R, correlation coefficient. eGFR, estimated glomerular filtration rate; uACR, urinary albumin‐to‐creatinine ratio

Figure 3 shows the correlation analysis results between eGFR, uACR and pulsatile stress. The pulsatile stress in childhood and eGFR in adults shown no correlation, regardless of sex (all *p *> .05). Adult uACR values were significantly correlated with pulsatile stress in childhood (*R *= 0.065, *p *= .008). In male patients, uACR values were significantly correlated with pulsatile stress in childhood (*R *= 0.095, *p *= .004), but the correlation was not found in female patients (*R *= 0.028, *p *= .445).

**FIGURE 4:**
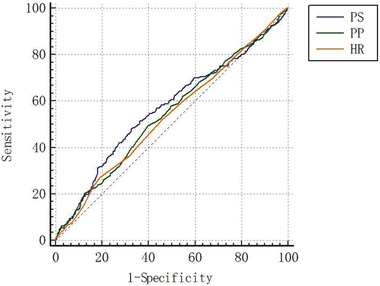
The receiver operator curve of predictive value of pulsatile stress, pulse pressure and heart rate for subclinical renal damage. HR, heart rate; PS, pulsatile stress; PP, pulse pressure

We used multivariable adjusted logistic regression to examine the association of pulsatile stress in childhood with SRD in adults (Table 2). The model adjusted for age, sex (for all patients), BMI and busts at baseline, BMI, waist, hips, smoking, drinking, fasting glucose, serum uric acid, triglycerides, total cholesterol, HDL‐C and LDL‐C at follow‐up. After further adjustment for pulsatile stress in adults, the HPS in childhood was still positively associated with adult SRD (RR, 1.43; 95%CI, 1.03–1.99). Specifically, HPS in childhood was associated with adult SRD in males (RR, 1.92; 95%CI, 1.26–2.94), but this association was not found in females (RR, 0.91; 95%CI, 0.52–1.58). Furthermore, we compared the predictive value of pulsatile stress, pulse pressure and heart rate in childhood for long‐term SRD by the area under the receiver operating curve (ROC). As shown in Figure 4, we found that the R^2^ value of pulsatile stress for predicting SRD was higher than R^2^ value of PP (0.568 vs. 0.545, *p *= .023) and heart rate (0.568 vs. 0.536, *p *= .031) for predicting SRD.

**TABLE 2:** Association of pulsatile stress in childhood and subclinical renal damage in adults, overall and by sex group

	n (%)	RR	95% CI	*p* value
All patients	221 (12.7%)			
Model 1		1.67	1.24–2.26	.001
Model 2		1.64	1.19–2.26	.003
Model 3		1.43	1.03–1.99	.032
Male patients	134 (13.9%)			
Model 1		1.99	1.35–2.94	<.001
Model 2		2.10	1.38–3.19	.001
Model 3		1.92	1.26–2.94	.003
Female patients	87 (11.2%)			
Model 1		1.29	0.79–2.11	.311
Model 2		1.11	0.66–1.87	.700
Model 3		0.91	0.52–1.58	.729

Model 1 was unadjusted; Model 2 was adjusted for age, sex (for all patients), body mass index and busts at baseline, body mass index, waist, hips, smoking, drinking, fasting glucose, serum uric acid, triglycerides, total cholesterol, high‐density lipoprotein cholesterol and low‐density lipoprotein cholesterol at follow‐up based on Model 1; Model 3 was adjusted for pulsatile stress at follow‐up based on Model 2. *n* (%), the number of individuals with SRD (%).

### 3.3 Association between pulsatile stress groups and SRD in adults

Furthermore, for the comparison of outcomes, the participants were categorized into four groups on the basis of pulsatile stress status in childhood and adulthood. Group I included participants with NPS in childhood and adulthood; group II, those who had NPS in childhood but HPS as adults; group III, those who had HPS in childhood but NPS as adults; and group IV, those with HPS in childhood and adulthood.

We used a logistic regression to examine the association between pulsatile stress groups and adult SRD. Age, sex, BMI and busts at baseline, BMI, waist, hips, smoking, drinking, fasting glucose, serum uric acid, triglycerides, total cholesterol, HDL‐C and LDL‐C at follow‐up were all adjusted. As shown in Table 3, compared with group I, group II (RR, 2.32; 95%CI, 1.58–3.40) and group IV (RR, 3.41; 95%CI, 2.16–5.39) had higher risks of adult SRD, except for group III (RR, 1.53; 95%CI, 0.99–2.35). We further classified all patients as male and female. In female patients, compared with group I, group II (RR, 2.68; 95%CI, 1.50–4.79) and group IV (RR, 2.99; 95%CI, 1.44–6.23) had higher risks of adult SRD, except for group III (RR, 0.96; 95%CI, 0.46–1.99). In male patients, with group I as reference, group II (RR, 2.09; 95%CI, 1.24–3.52), group III (RR, 2.04; 95%CI, 1.18–3.54) and group IV (RR, 3.77; 95%CI, 2.07–6.89) all had higher risks of adult SRD.

**TABLE 3:** Relative risks of subclinical renal damage in adults according to pulsatile stress group in childhood and adulthood

		Unadjusted model		Adjusted MODEL	
	n (%)	RR (95% CI)	*p* value	RR (95% CI)	*p* value
All patients					
Group I	86 (8.5%)	Reference		Reference	
Group II	59 (20.6%)	2.79 (1.94, 4.01)	<.001	2.32 (1.58, 3.40)	<.001
Group III	36 (12.1%)	1.49 (0.98, 2.25)	.060	1.53 (0.99, 2.35)	.053
Group IV	40 (28.4%)	4.27 (2.78, 6.55)	<.001	3.41 (2.16, 5.39)	<.001
Male patients					
Group I	52 (9.2%)	Reference		Reference	
Group II	32 (20.5%)	2.55 (1.58, 4.13)	<.001	2.09 (1.24, 3.52)	.006
Group III	25 (15.6%)	1.83 (1.10, 3.06)	.021	2.04 (1.18, 3.54)	.011
Group IV	25 (30.9%)	4.41 (2.54, 7.66)	<.001	3.77 (2.07, 6.89)	<.001
Female patients					
Group I	34 (7.6%)	Reference		Reference	
Group II	27 (20.6%)	3.15 (1.82, 5.46)	<.001	2.68 (1.50, 4.79)	.001
Group III	11 (8.0%)	1.06 (0.52, 2.15)	.871	0.96 (0.46, 1.99)	.911
Group IV	15 (25.0%)	4.05 (2.05, 8.00)	<.001	2.99 (1.44, 6.23)	.003

Adjusted Model was adjusted for age, sex (for all patients), body mass index and busts at baseline, body mass index, waist, hips, smoking, drinking, fasting glucose, serum uric acid, triglycerides, total cholesterol, high‐density lipoprotein cholesterol and low‐density lipoprotein cholesterol at follow‐up. *n* (%), the number of individuals with SRD (%).

### 3.4 Sensitivity analyses

We performed additional analyses after excluding individuals who had diabetes or received antihypertensive drugs (*n* = 82) to eliminate the effect of these factors on the results. As shown in Tables S2 and S3, the results of the sensitivity analyses remained the same. Furthermore, we performed a sensitivity analysis after excluding individuals with eGFR > 200 ml/min per 1.73 m^2^ (*n* = 10), and the results remained the same (Table S4). We performed an additional analysis of the relationship between pulsatile stress and subclinical renal damage using a threshold of 30 mg/g of uACR, and the results remained the same (Table S5). In addition, we also performed an analysis of the relationship between pulsatile stress and renal damage by adjusted BMI z‐score rather than BMI at baseline. The results of the two are consistent (Table S6).

## 4 DISCUSSION

Our analysis included 1738 schoolchildren aged 6–15 years who were enrolled from rural China in 1987 and followed for 30 years. In this study, we assessed the association between pulsatile stress in childhood and adult subclinical renal damage in an ongoing adolescent prospective cohort. We found that pulsatile stress in childhood was significantly associated with adult SRD in males, but this association has not been found in females. In addition, we demonstrated that HPS in childhood, completely independent of adult pulsatile pressure, can increased the risk of adult SRD in males, while female patients did not. In other words, for male individuals, regardless of their adult pulsatile stress, childhood HPS will increase the risk of SRD in middle age.

CKD is associated with increased risks of all‐cause mortality, cardiovascular disease, and progression to kidney failure.^20^, ^21^ In a cross‐sectional pediatric chronic kidney disease cohort from the CKiD study, they found that the pulse pressure was associated with lower eGFR in pediatric CKD patients.^22^ A community‐based cohort study included 1426 participants, followed for a median of 4.8 years and found that central pulse pressure is an independent determinant of renal function.^23^ And previous study have demonstrated that high resting heart rate is a potent predictor of renal outcomes.^24^ However, there is no prospective data confirming whether pulsatile stress (the combined effect of the two components) in early life affect renal function in middle age. In our study, we investigated the association between pulsatile stress in childhood and adult SRD using an ongoing adolescent prospective cohort. We found the prevalence of adult SRD in HPS group was higher than NPS group. And adolescents with HPS were 1.4 times more likely to have SRD in middle life than normal individuals. And we found that the predictive value of pulsatile stress, that is, combined pulse pressure and heart rate, for SRD is higher than any one of them alone. Our results were similar with a kidney transplant study. They revealed an eightfold increased risk for microalbuminuria and 12.2‐fold increased risk for macro‐albuminuria comparing upper with lower tertile of pulsatile stress.^25^ Our study, while different, emphasize the effect of childhood pulsatile stress on adult SRD. In this context, pulse pressure may alone not be relevant for the determination of the microcirculatory damage. Other parameters may be needed, such as increased sympathetic activity, which accelerates heart rate. After combining pulse pressure and heart rate, continuously enhanced pulsatility down to microcirculatory level may thus distort resistance arteries, which can directly influence intraglomerular pressure and flow conditions.^25^, ^26^

Further, we found a sex difference in the association between childhood pulsatile stress and SRD in adults. We found that HPS in childhood can increase the risk of adult SRD in males, but this association has not been found in females. Similarly, we revealed that HPS in childhood, independent of adult pulsatile pressure, had 2.0‐fold increased risk for adult SRD in males, while female patients did not. Consistent with our findings, a large French population study demonstrated a combined elevation of the two components of pulsatile arterial stress was associated with an important increase in cardiovascular mortality in men.^10^ McEniery and coworkers also found pulse pressure heart rate product assessed over the whole of 20 years was independently correlated with aortic pulse wave velocity in males.^27^ The mechanism for this sex difference may be that both pulse pressure and heart rate play a more important role in men than in women.^10^, ^28^

We also found that childhood pulsatile stress was associated with adult uACR levels but not eGFR. Our results are in line with a finding we have already reported in a previous study that higher BP trajectories were correlated with higher of uACR levels but not eGFR.^14^ The failure to reach significance for eGFR was mainly due to the small number of patients with abnormal eGFR range in our study. In addition, the difference between uACR and eGFR may be caused by various characteristics of the analyzed population, such as age. The eGFR values was used to define kidney damage, severe CKD, and end‐stage renal disease. As Huang and coworkers has revealed, in old people, aortic stiffness was significantly associated with a decrease in eGFR, but not with uACR levels.^29^ On the contrary, micro‐albuminuria is an accepted marker for microcirculatory damage,^30^ more appropriate in young and middle‐aged individuals. In our study, patients were recruited in childhood and followed for 30 years to middle age, and the renal function was assessed by eGFR and uACR jointly to make the result more accurate.

There are several strengths in this study. First, this is a large number of adolescent cohort with the prospective follow up in nature, allowing us to fully explore risk factors in childhood. Second, individuals were followed for 30 years up to adulthood, with a relatively high response rate, which is sufficient to assess the impact of childhood variables on adult target organ damage. Third, this study focused on exploring the association between childhood pulsatile stress and adult SRD. To our knowledge, this impact has rarely been explored in previous studies. However, our study also has limitations. First of all, our study only included Han people in northern China. Therefore, epidemiological studies of other ethnicities are needed to verify our results. Second, there is a lack of data on physical activity, diet and other risk factors that may affect renal function in our cohort. Third, the eGFR value in this study was estimated based on serum creatinine. Although the value is calculated according to the internationally recognized standard formula, it is still relatively inaccurate. More accurate filtration markers, such as cystatin C or β2‐microglobulin, were not examined. Fourthly, the use of a single morning spot urine sample to evaluate uACR is also a major limitation of this study. However, in this study, every step from the collection, storage and detection of urine specimens was strictly and accurately completed, which can improve its stability and reduce the false positive rate. Finally, the mean age of the patients was 42 years old at the last follow‐up. Therefore, the prevalence of SRD is relatively low, especially the number of individuals with abnormal eGFR range. However, the prospective design of our study provides us an opportunity to perform further follow‐ups to determine the future risk of CKD.

In summary, this study demonstrated that high pulsatile stress in childhood significantly increased the risk of adult SRD, especially in males. Adequate control of pulse pressure and heart rate from childhood, in the long‐term, is very important for preventing kidney damage.

## CONFLICT OF INTEREST

None.

## Supporting information

Supplementary Information

Supplementary Information
