# Supplementary figures and images for: Association of pulsatile stress in childhood with subclinical renal damage in adults: A 30‐year prospective cohort study
Source: J Clin Hypertens (Greenwich). 2021 Sep 8;23(10):1843–51. doi: 10.1111/jch.14360 (PMC8678770; doi:10.1111/jch.14360)

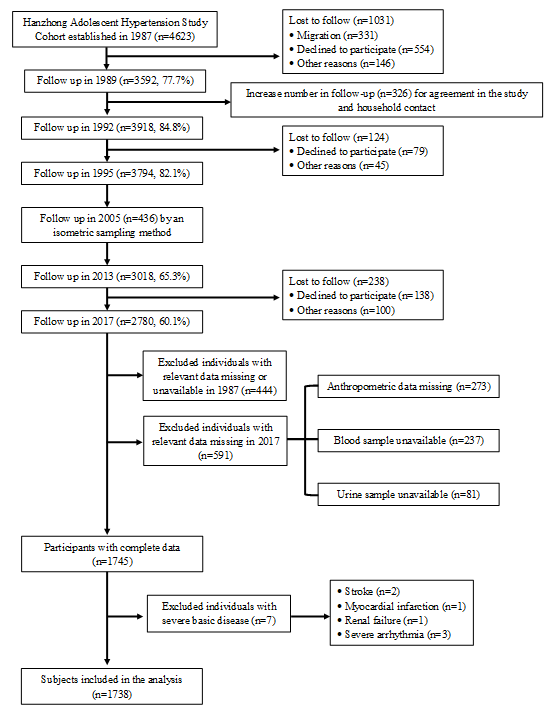

Supplement: Supplementary file 1 — Supplementary Information [file JCH-23-1843-s001.tif]
