# Supplementary material for: Association of pulsatile stress in childhood with subclinical renal damage in adults: A 30‐year prospective cohort study
Source: J Clin Hypertens (Greenwich). 2021 Sep 8;23(10):1843–51. doi: 10.1111/jch.14360 (PMC8678770; doi:10.1111/jch.14360)
Supplement: Supplementary file 2 — Supplementary Information [file JCH-23-1843-s002.docx]

| Supplemental Table 1. The Pulsatile stress quartiles of study participants in childhood According to the age categories. | | | | | |
| --- | --- | --- | --- | --- | --- |
| Age (years) | n (%) | 1st quartile group | 2nd quartile group | 3rd quartile group | 4th quartile group |
| 6 | 65 (3.74%) | 2452.0 (2197.2, 2612.0) | 2897.4 (2852.5, 2980.0) | 3264.0 (3183.2, 3549.6) | 4212.0 (3895.5, 4530.5) |
| 7 | 126 (7.25%) | 2346.0 (2074.5, 2431.8) | 2640.1 (2564.5, 2751.0) | 3090.6 (2949.0, 3284.0) | 3772.8 (3601.8, 3854.8) |
| 8 | 158 (9.09%) | 2210.4 (1989.0, 2286.7) | 2664.0 (2535.0, 2778.8) | 3126.6 (2987.4, 3249.2) | 3717.0 (3526.6, 4241.2) |
| 9 | 160 (9.21%) | 2057.7 (1934.0, 2196.4) | 2545.2 (2448.0, 2664.0) | 2984.0 (2888.4, 3131.1) | 3672.0 (3528.0, 4008.6) |
| 10 | 142 (8.17%) | 2028.0 (1757.6, 2220.0) | 2588.0 (2478.0, 2690.4) | 3029.9 (2902.9, 3125.7) | 3624.0 (3457.8, 4066.2) |
| 11 | 185 (10.64%) | 2160.0 (2016.0, 2271.2) | 2715.8 (2585.2, 2836.0) | 3088.0 (3029.4, 3192.0) | 3794.4 (3596.3, 4016.0) |
| 12 | 180 (10.36%) | 2160.0 (1896.0, 2274.4) | 2541.6 (2400.0, 2690.4) | 3007.8 (2880.0, 3120.0) | 3746.8 (3416.0, 3952.0) |
| 13 | 228 (13.12%) | 2210.4 (2034.0, 2324.9) | 2750.7 (2611.0, 2856.0) | 3229.6 (3096.0, 3405.6) | 3896.0 (3696.0, 4276.8) |
| 14 | 274 (15.77%) | 2285.4 (1967.2, 2393.8) | 2849.0 (2736.0, 2941.2) | 3248.0 (3143.4, 3385.2) | 3900.0 (3744.0, 4480.0) |
| 15 | 220 (12.66%) | 2280.0 (2059.2, 2448.0) | 2909.4 (2776.0, 3000.0) | 3410.4 (3258.0, 3576.0) | 4212.0 (3855.0, 4582.5) |
| 1st quartile, the first quartile; 2nd quartile, the second quartile; 3rd quartile, the third quartile; 4th quartile, the fourth quartile. | | | | | |

| Supplemental Table 2. Association of Pulsatile Stress in childhood and Subclinical Renal Damage in adults, Overall and by Sex Group (Sensitivity analysis). | | | | |
| --- | --- | --- | --- | --- |
|  | n (%) | RR | 95% CI | *P* Value |
| All Subjects | 187 (11.3%) |  |  |  |
| Model 1 |  | 1.73 | 1.25 - 2.39 | 0.001 |
| Model 2 |  | 1.69 | 1.20 - 2.36 | 0.002 |
| Model 3 |  | 1.46 | 1.03 - 2.07 | 0.032 |
| Male Subjects | 109 (12.0%) |  |  |  |
| Model 1 |  | 2.21 | 1.46 - 3.35 | <0.001 |
| Model 2 |  | 2.16 | 1.39 - 3.35 | 0.001 |
| Model 3 |  | 1.98 | 1.26 - 3.09 | 0.003 |
| Female Subjects | 78 (10.5%) |  |  |  |
| Model 1 |  | 1.21 | 0.72 - 2.04 | 0.481 |
| Model 2 |  | 1.15 | 0.67 - 1.98 | 0.616 |
| Model 3 |  | 0.90 | 0.50 - 1.60 | 0.707 |
| Model 1 was unadjusted; Model 2 was adjusted for age, sex (for all subjects), body mass index and busts at baseline, body mass index, waist, hips, smoking, drinking, fasting glucose, serum uric acid, triglycerides, total cholesterol and low-density lipoprotein cholesterol at follow-up based on Model 1; Model 3 was adjusted for pulsatile stress at follow-up based on Model 2. n (%), the number of individuals with SRD (%). | | | | |

| Supplemental Table 3. Relative Risks of Subclinical Renal Damage in adults According to Pulsatile Stress Group in Childhood and Adulthood (Sensitivity analysis). | | | | | |
| --- | --- | --- | --- | --- | --- |
|  |  | Unadjusted Model | | Adjusted Model | |
|  | n (%) | RR (95% CI) | *P* value | RR (95% CI) | *P* value |
| All Subjects |  |  |  |  |  |
| Group Ⅰ | 73 (7.5%) | Reference |  | Reference |  |
| Group Ⅱ | 48 (18.3%) | 2.76 (1.86, 4.09) | <0.001 | 2.40 (1.60, 3.62) | <0.001 |
| Group Ⅲ | 32 (11.1%) | 1.57 (1.01, 2.47) | 0.048 | 1.54 (0.99, 2.38) | 0.054 |
| Group Ⅳ | 34 (26.4%) | 4.42 (2.80, 6.70) | <0.001 | 3.68 (2.28, 5.96) | <0.001 |
| Male Subjects |  |  |  |  |  |
| Group Ⅰ | 40 (7.5%) | Reference |  | Reference |  |
| Group Ⅱ | 25 (17.5%) | 2.62 (1.53, 4.49) | <0.001 | 2.16 (1.23, 3.80) | 0.008 |
| Group Ⅲ | 22 (14.2%) | 2.05 (1.18, 3.56) | 0.011 | 2.15 (1.20, 3.83) | 0.010 |
| Group Ⅳ | 22 (28.6%) | 4.95 (2.74, 8.93) | <0.001 | 3.87 (2.05, 7.30) | <0.001 |
| Female Subjects | |  |  |  |  |
| Group Ⅰ | 33 (7.5%) | Reference |  | Reference |  |
| Group Ⅱ | 23 (19.2%) | 2.92 (1.64, 5.21) | <0.001 | 2.71 (1.48, 4.98) | 0.001 |
| Group Ⅲ | 10 (7.5%) | 0.99 (0.48, 2.08) | 0.989 | 0.97 (0.46, 2.05) | 0.936 |
| Group Ⅳ | 12 (23.1%) | 3.70 (1.77, 7.73) | <0.001 | 3.37 (1.56, 7.28) | 0.002 |
| Adjusted Model was adjusted for age, sex (for all subjects), body mass index and busts at baseline, body mass index, waist, hips, smoking, drinking, fasting glucose, serum uric acid, triglycerides, total cholesterol and low-density lipoprotein cholesterol at follow-up. n (%), the number of individuals with SRD (%). | | | | | |

| Supplemental Table 4. Association of Pulsatile Stress in childhood and Subclinical Renal Damage in adults after excluding individuals with eGFR > 200 ml/min per 1.73 m^2^ (n=10), Overall and by Sex Group. | | | | |
| --- | --- | --- | --- | --- |
|  | n (%) | RR | 95% CI | *P* Value |
| All Subjects | 218 (12.5%) |  |  |  |
| Model 1 |  | 1.64 | 1.21 - 2.22 | 0.002 |
| Model 2 |  | 1.49 | 1.06 - 2.11 | 0.022 |
| Model 3 |  | 1.36 | 1.01 - 1.94 | 0.044 |
| Male Subjects | 131 (13.7%) |  |  |  |
| Model 1 |  | 1.94 | 1.31 - 2.86 | 0.001 |
| Model 2 |  | 1.97 | 1.26 - 3.08 | 0.003 |
| Model 3 |  | 1.86 | 1.18 - 2.93 | 0.007 |
| Female Subjects | 87 (11.3%) |  |  |  |
| Model 1 |  | 1.28 | 0.78 - 2.09 | 0.326 |
| Model 2 |  | 0.95 | 0.54 - 1.67 | 0.863 |
| Model 3 |  | 0.83 | 0.46 - 1.49 | 0.527 |
| Model 1 was unadjusted; Model 2 was adjusted for age, sex (for all subjects), body mass index and busts at baseline, body mass index, waist, hips, smoking, drinking, fasting glucose, serum uric acid, triglycerides, total cholesterol, high-density lipoprotein cholesterol and low-density lipoprotein cholesterol at follow-up based on Model 1; Model 3 was adjusted for pulsatile stress at follow-up based on Model 2. n (%), the number of individuals with SRD (%). | | | | |
| Supplemental Table 5. Association of Pulsatile Stress in childhood and Subclinical Renal Damage in adults, Overall and by Sex Group (sensitivity analysis). | | | | |
|  | n (%) | RR | 95% CI | *P* Value |
| All Subjects | 186 (10.7%) |  |  |  |
| Model 1 |  | 1.48 | 1.07 - 2.05 | 0.020 |
| Model 2 |  | 1.40 | 1.03 - 1.99 | 0.045 |
| Model 3 |  | 1.21 | 1.01 - 1.72 | 0.049 |
| Male Subjects | 94 (9.8%) |  |  |  |
| Model 1 |  | 1.85 | 1.16 - 2.84 | 0.009 |
| Model 2 |  | 1.81 | 1.14 - 2.99 | 0.012 |
| Model 3 |  | 1.67 | 1.03 - 2.72 | 0.039 |
| Female Subjects | 92 (11.9%) |  |  |  |
| Model 1 |  | 1.18 | 0.73 - 1.92 | 0.505 |
| Model 2 |  | 1.02 | 0.61 - 1.72 | 0.930 |
| Model 3 |  | 0.82 | 0.47 - 1.42 | 0.472 |
| Model 1 was unadjusted; Model 2 was adjusted for age, sex (for all subjects), body mass index and busts at baseline, body mass index, waist, hips, smoking, drinking, fasting glucose, serum uric acid, triglycerides, total cholesterol, high-density lipoprotein cholesterol and low-density lipoprotein cholesterol at follow-up based on Model 1; Model 3 was adjusted for pulsatile stress at follow-up based on Model 2. n (%), the number of individuals with SRD (%). | | | | |

| Supplemental Table 6. Association of Pulsatile Stress in childhood and Subclinical Renal Damage in adults, Overall and by Sex Group (Additional analysis). | | | | |
| --- | --- | --- | --- | --- |
|  | n (%) | RR | 95% CI | *P* Value |
| All Subjects | 221 (12.7%) |  |  |  |
| Model 1 |  | 1.67 | 1.24 - 2.26 | 0.001 |
| Model 2 |  | 1.63 | 1.18 - 2.25 | 0.003 |
| Model 3 |  | 1.42 | 1.03 - 1.98 | 0.034 |
| Male Subjects | 134 (13.9%) |  |  |  |
| Model 1 |  | 1.99 | 1.35 - 2.94 | <0.001 |
| Model 2 |  | 2.10 | 1.38 - 3.20 | <0.001 |
| Model 3 |  | 1.92 | 1.26 - 2.94 | 0.003 |
| Female Subjects | 87 (11.2%) |  |  |  |
| Model 1 |  | 1.29 | 0.79 - 2.11 | 0.311 |
| Model 2 |  | 1.11 | 0.66 - 1.87 | 0.703 |
| Model 3 |  | 0.91 | 0.52 - 1.57 | 0.726 |
| Model 1 was unadjusted; Model 2 was adjusted for age, sex (for all subjects), BMI z-score and busts at baseline, body mass index, waist, hips, smoking, drinking, fasting glucose, serum uric acid, triglycerides, total cholesterol, high-density lipoprotein cholesterol and low-density lipoprotein cholesterol at follow-up based on Model 1; Model 3 was adjusted for pulsatile stress at follow-up based on Model 2. n (%), the number of individuals with SRD (%). | | | | |

Supplemental Figure 1: Flow of participants.
